# Risk Factors for Cardiovascular-Specific Mortality in Patients With Prostate Cancer: A Surveillance, Epidemiology, and End Results (SEER)-Based Study

**DOI:** 10.7759/cureus.51279

**Published:** 2023-12-29

**Authors:** Abdulhameed Alhadeethi, Ahmed Atia, Ibraheem M Alkhawaldeh, Ahmed A Ibrahim, Eslam Afifi, Ahmed Elwekel, Abdallah Nouh, Maha H Morsi

**Affiliations:** 1 General Medicine, Al-Salam Teaching Hospital, Mosul, IRQ; 2 General Medicine, Medical Research Group of Egypt, Negida Academy LCC, Arlington, USA; 3 Oncology, Faculty of Medicine Kasr Al-Ainy, Cairo University, Cairo, EGY; 4 Cardiology, Faculty of Medicine, Mu'tah University, Karak, JOR; 5 Cardiology, Faculty of Medicine, Menoufia University, Shibin El Kom, EGY; 6 Urology, Faculty of Medicine, Benha University, Benha, EGY; 7 Cardiology, Faculty of Medicine, Al-Azhar University, Cairo, EGY; 8 Oncology, Faculty of Medicine, Sohag University, Sohag, EGY; 9 Urology, Misr University for Science and Technology, 6th of October City, EGY

**Keywords:** risk factors, prostate cancer (pc), survival analysis, national cancer database and seer analyses, cardiovascular-related mortality

## Abstract

Background: Prostate cancer (PC) is responsible for large numbers of cancer-related deaths in males worldwide, and it has been linked to an increase in cardiovascular morbidity and mortality (CVM). The purpose of this research is to identify the incidence and risk factors for CVM in PC patients.

Methods: In ﻿this retrospective cohort study, we collected data from patients with PC diagnosed between 2000 and 2014 from the Surveillance, Epidemiology, and End Results (SEER) database. CVM among PC patients was identified and compared to the general population using the standardized mortality ratio (SMR). The multivariable competing risk model with subdistribution hazard ratio (SHR) was used to analyze the data in a more complex method to discover the risk factors associated with CVM among PC patients.

Results: Of the 171,147 identified PC patients, the median survival time was 117 months, with 17,168 dying from cardiovascular disease (CVD). Patients diagnosed at age 45-54 had a higher CVM risk than the age-standardized general population (SMR (95% CI): 19.01 (17.17-21.0)). Using multivariate competing risk regression analysis, aged 85 and older (SHR (95% CI): 20.9 (18.628-23.467)), black ethnicities (SHR (95% CI): 1.3 (1.264-1.398)), and patients without surgical intervention (SHR (95% CI): 1.35 (1.305-1.410)) had higher CVM. On the other hand, being of Asian/Pacific Islander or American Indian/Native Alaskan ethnicity (SHR (95% CI): 0.94 (0.891-0.993)), being diagnosed between 2007 and 2014 (SHR (95% CI): 0.63 (0.613-0.655)), and having an advanced disease stage and a lack of disease differentiation in the histology were found to be related with a lower CVM.

Conclusion: Patients with PC have a greater likelihood of dying from CVD. Several important risk factors for CVD have been discovered, including advanced age, black ethnicity, and patients without surgical intervention. These findings are limited by the retrospective nature of the analysis, relying solely on the SEER database, which imposes restrictions on accessing comprehensive patient data, including lifestyle factors and medical history.

## Introduction

Prostate cancer (PC) accounts for nearly one-fifth of cancer-related deaths in male patients worldwide [1]. This contributed to approximately 34,500 deaths in 2022 in the USA (around 5.7% of all cancer deaths) and 10.2 per 100,000 in Australia and New Zealand [2,3]. The incidence of PC is high among African-American ethnicities [4]. PC has been associated with many well-established risk factors, such as old age, family history, high dietary intake of saturated animal fat and red meat, and low dietary intake of fruits and vegetables [5]. In addition, low physical activity, obesity, inflammation, and hyperglycemia are risk factors positively associated with the incidence of PC [2].

Cardiovascular disease (CVD) mortality remains the primary cause of death worldwide. The prevalence of CVD cases has doubled chiefly from 1990 to 2019 [6]. There are many risk factors for CVD, like alcohol, smoking, ultra-processed foods, and cancer [7,8]. Today, our cancer treatments and medical care have improved, increasing life expectancy, but comorbidities have also increased, with CVD being the leading cause of death in cancer patients [9]. In addition, people who have survived cancer are more likely to experience CVD events, because of either the cancer treatment or the cancer itself [8].

PC was highly associated with the development of cardiovascular comorbidities and mortality [10]. Compared to the general population, PC survivors had a higher rate of cardiovascular risk factors that could lead to death [11,12]. Androgen deprivation therapy (the medical treatment of PC) increases the risk of insulin resistance, dyslipidemia, and obesity, leading to an increase in cardiovascular-specific events like stroke, myocardial infarction, and acute coronary syndrome [13,14]. Also, orchiectomy (the surgical treatment for PC) showed the same risk [13]. Because of these risk factors, CVD is likely to significantly affect the quality of life of people who have had PC [11].

In contrast, exercise was found to improve cardiorespiratory fitness and inhibit PC progression [10]. To the best of our knowledge, the available data and research studies addressing the relationship between primary PC and the risk of death from CVDs are limited. So, the objective of this study was to determine the incidence of CVD-specific mortality, as well as which factors have the greatest impact on the risk of death specifically due to CVD in this patient population.

This article has been accepted to be presented as a poster at the 15th European Multidisciplinary Congress on Urological Cancers (EMUC23) Annual Scientific Congress in November 2023.

## Materials and methods

﻿Data sources and study design

We conducted a retrospective analysis of the prospectively collected database following the Strengthening the Reporting of Observational Studies in Epidemiology (STROBE) reporting guideline [15]. We used the Surveillance, Epidemiology, and End Results (SEER) database to retrieve the data of PC patients diagnosed in the period of 2000-2014 using SEER*Stat software (National Cancer Institute, Bethesda, Maryland, United States) [16] through Incidence - SEER Research Data, 8 Registries, Nov 2021 Sub (1975-2019) [17]. The expected number of deaths derived for each of the subgroups was obtained from the Centers for Disease Control and Prevention Wide-Ranging Online Data for Epidemiologic Research (CDC WONDER) [18].

Eligibility criteria

**Figure 1:**
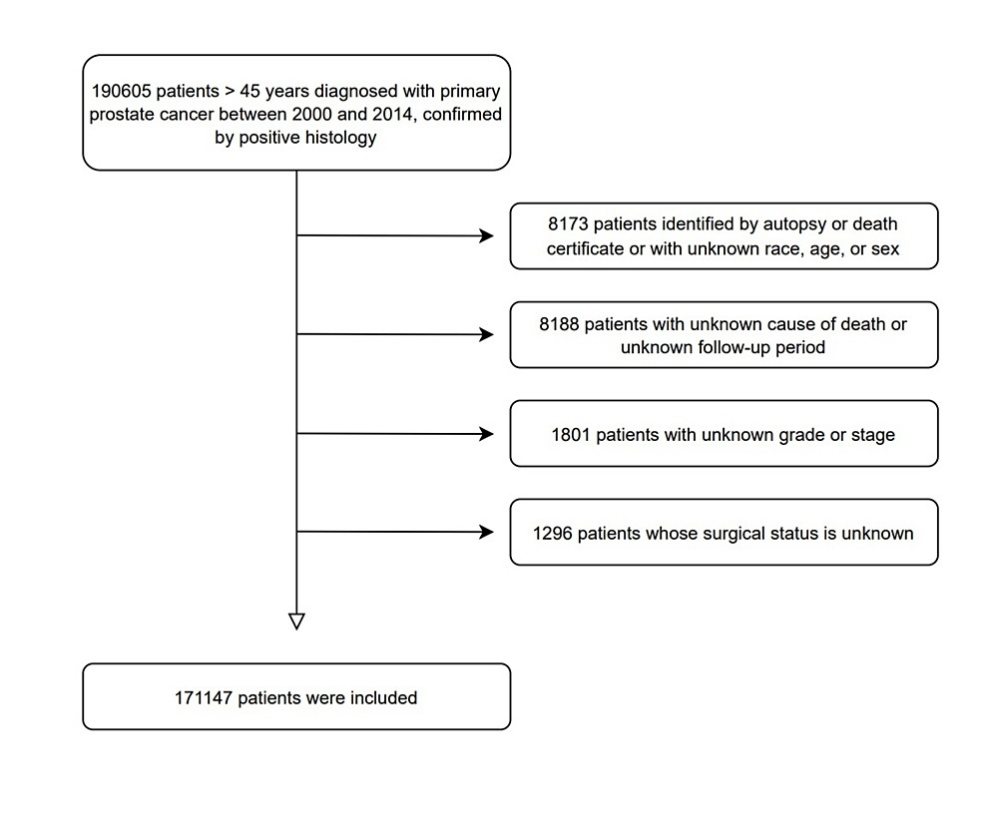
Flow diagram showing the inclusion and exclusion criteria

We included patients older than 45 years with only one primary PC confirmed by histology. The exclusion criteria were as follows: (1) no positive histology; (2) unknown cause of death; (3) unknown age, race, and sex; (4) unknown grade; (5) unknown follow-up duration; (6) unknown state of surgical intervention; and (7) identified by autopsy or death certificate. The details of patients' eligibility are shown in Figure 1.

﻿Study variables

The primary outcome of interest was CVM concerning the following collected data: age at diagnosis (45-54, 55-64, 65-74, 75-84, 85+ years), race (white, black, others), year of diagnosis (2000-2006, 2007-2014), surgical intervention, tumor grade, and summary stage (localized, regional, distant). Follow-up was terminated upon the occurrence of the primary endpoint event or the conclusion of follow-up ﻿(December 31, 2014). Causes of death have been reported in the SEER database according to the International Classification of Diseases (ICD-10). CVM was defined as death caused by diseases of the heart, atherosclerosis (I70), cerebrovascular diseases (I60-I69), hypertension without heart disease (I10, I12), other diseases of the arteries, arterioles, and capillaries (I72-I78), and aortic aneurysm and dissection (I71).

Statistical analysis

In this study, we used the chi-squared test to assess the relationship between the dichotomous variables. Additionally, standardized mortality ratios (SMRs) with﻿ 95% confidence intervals were used to compare the mortality rates between the study population and the general population. This helped determine if the study population was at a higher or lower risk of death compared to the general population. Furthermore, a multivariable competing risk model with subdistribution hazard ratio (SHR) was used to analyze the data in a more complex manner [19]. This model considers multiple variables and their interactions, and it allows for the analysis of subdistribution hazards. The SHR answers questions about incidence and prognosis, which can provide a more accurate representation of the data. All analyses were conducted using Jamovi (https://www.jamovi.org/), Version 2.3 [20-23], and Stata/MP (https://www.stata.com/statamp/), Version 16.0. The SMR was calculated using OpenEpi (www.openepi.com), Version 3. The significance level was set at 0.05.

## Results

Characteristics of the study population

According to our inclusion criteria, there were 171,147 patients diagnosed with PC between 2000 and 2014 in the SEER database, of whom 54,305 (31.7%) passed away during the follow-up period with a median survival time of 117 months, including 17,168 patients who died from CVD and 14,272 deaths from PC. Most patients who died from CVD were aged between 65 and 74 years at diagnosis (37.5%), were of white ethnicity (81.2%), and had a moderately differentiated disease on histology (51.4%). Moreover, 84.5% had localized disease, with 51.8% having not undergone surgery. Table 1 shows the baseline characteristics.

**Table 1:** Characteristics of the study population by causes of death ^1^Pearson's chi-squared test CVD: cardiovascular diseases; 85+: age 85 or older; others: Asian/Pacific Islander or American Indian/Native Alaskan ethnicity

Age groups (years)	Alive (N=116,842)	CVD (N=17,168)	Prostate cancer (N=14,272)	Other causes (N=22,865)	Total (N=171,147)	p-value
							45-54	16,634 (14.2%)	379 (2.2%)	981 (6.9%)	637 (2.8%)	18,631 (10.9%)	<0.001^1^
55-64	49,687 (42.5%)	2,356 (13.7%)	3,278 (23.0%)	3,390 (14.8%)	58,711 (34.3%)	
65-74	41,306 (35.4%)	6,438 (37.5%)	4,693 (32.9%)	8,935 (39.1%)	61,372 (35.9%)	
75-84	8,847 (7.6%)	6,351 (37.0%)	4,099 (28.7%)	8,211 (35.9%)	27,508 (16.1%)	
85+	368 (0.3%)	1,644 (9.6%)	1,221 (8.6%)	1,692 (7.4%)	4,925 (2.9%)	
Race						
White	95,217 (81.5%)	13,941 (81.2%)	11,466 (80.3%)	18,809 (82.3%)	139,433 (81.5%)	<0.001^1^
Black	13,206 (11.3%)	1,791 (10.4%)	1,747 (12.2%)	2,246 (9.8%)	18,990 (11.1%)	
Others	8,419 (7.2%)	1,436 (8.4%)	1,059 (7.4%)	1,810 (7.9%)	12,724 (7.4%)	
Year of diagnosis						
2000-2006	40,960 (35.1%)	11,877 (69.2%)	8,357 (58.6%)	15,426 (67.5%)	76,620 (44.8%)	<0.001^1^
2007-2014	75,882 (64.9%)	5,291 (30.8%)	5,915 (41.4%)	7,439 (32.5%)	94,527 (55.2%)	
Grade						
Well differentiated	4,274 (3.7%)	411 (2.4%)	84 (0.6%)	524 (2.3%)	5,293 (3.1%)	<0.001^1^
Moderately differentiated	60,057 (51.4%)	9,004 (52.4%)	2,947 (20.6%)	11,981 (52.4%)	83,989 (49.1%)	
Poorly differentiated	52,363 (44.8%)	7,704 (44.9%)	11,086 (77.7%)	10,290 (45.0%)	81,443 (47.6%)	
Undifferentiated	148 (0.1%)	49 (0.3%)	155 (1.1%)	70 (0.3%)	422 (0.2%)	
Surgery						
Performed	56,260 (48.2%)	4,240 (24.7%)	3,456 (24.2%)	5,955 (26.0%)	69,911 (40.8%)	<0.001^1^
Not performed	60,582 (51.8%)	12,928 (75.3%)	10,816 (75.8%)	16,910 (74.0%)	101,236 (59.2%)	
Summary stage						
Localized	98,721 (84.5%)	15,257 (88.9%)	7,346 (51.5%)	20,196 (88.3%)	141,520 (82.7%)	<0.001^1^
Regional	17,267 (14.8%)	1,397 (8.1%)	2,509 (17.6%)	1,999 (8.7%)	23,172 (13.5%)	
Distant	854 (0.7%)	514 (3.0%)	4,417 (30.9%)	670 (2.9%)	64,550 (3.8%)	

SMRs

Patients diagnosed at ages 45-54 had a greater CVM risk compared to the age-standardized general population (SMR (95% CI): 13.58 (12.27-15.01)), while patients diagnosed at ages 85 or above had the lowest CVM risk (SMR (95% CI): 4.983 (4.747-5.229)). Also, we evaluated the mortality risk of CVD among different ethnic groups. The results showed that the SMR for white ethnicity was SMR (95% CI): 13.43 (13.2-13.65), for black ethnicity, it was SMR (95% CI): 11.55 (11.02-12.09), and for Indian/Native Alaskan or Asian/Pacific Islander ethnicity, it was SMR (95% CI): 31.35 (29.76-33.01). This is more detailed in Table 2.

**Table 2:** SMR for cardiovascular deaths among prostate cancer patients SMR: standardized mortality ratio; 85+: age 85 or older; others: Asian/Pacific Islander or American Indian/Native Alaskan ethnicity

Age group (years)	﻿Observed deaths	﻿Expected deaths	SMR	﻿95% CI lower	﻿95% CI upper
45-54	379	27.9	13.58	12.27	15.01
55-64	2,356	207.2	11.37	10.92	11.84
65-74	6,438	492.3	13.08	12.76	13.4
75-84	6,351	605.3	10.49	10.24	10.75
85+	1,644	329.9	4.983	4.747	5.229
Race					
White	13,941	1,038.4	13.43	13.2	13.65
Black	1,791	155.11	11.55	11.02	12.09
Others	1,436	45.8	31.35	29.76	33.01

Death indicators associated with CVD mortality

In patients with PC, factors associated with the incidence of CVM events were identified using the Fine-Gray multivariate competing risk regression analysis. This is shown in Table 3.

**Table 3:** Multivariate competing risk regression analysis of CVD mortality in patients with prostate cancer SHR: subdistribution hazard ratio; CI: confidence interval; CVD: cardiovascular disease; 85+: age 85 or older; others: Asian/Pacific Islander or American Indian/Native Alaskan ethnicity

Age group (years)	SHR	﻿95% CI	p-value
45-54	Ref		
55-64	2.03	1.821-2.26	0.001
65-74	5.21	4.69-5.78	0.001
75-84	11.37	10.23-12.64	0.001
85+	20.90	18.62-23.46	0.001
Race			
White	Ref		
Black	1.32	1.26-1.39	0.001
Others	0.94	0.89-0.99	0.029
Year of diagnosis			
2000-2006	Ref		
2007-2014	0.63	0.61-0.65	0.001
Grade			
Well differentiated	Ref		
Moderately differentiated	0.89	0.80-0.98	0.023
Poorly differentiated	0.87	0.79-0.97	0.011
Undifferentiated	0.84	0.62-1.15	0.286
Surgery performed			
Yes	Ref		
No	1.35	1.30-1.41	0.001
Summary stage			
Localized	Ref		
Regional	0.90	0.85-0.96	0.001
Distant	0.54	0.50-0.60	0.001

**Figure 2:**
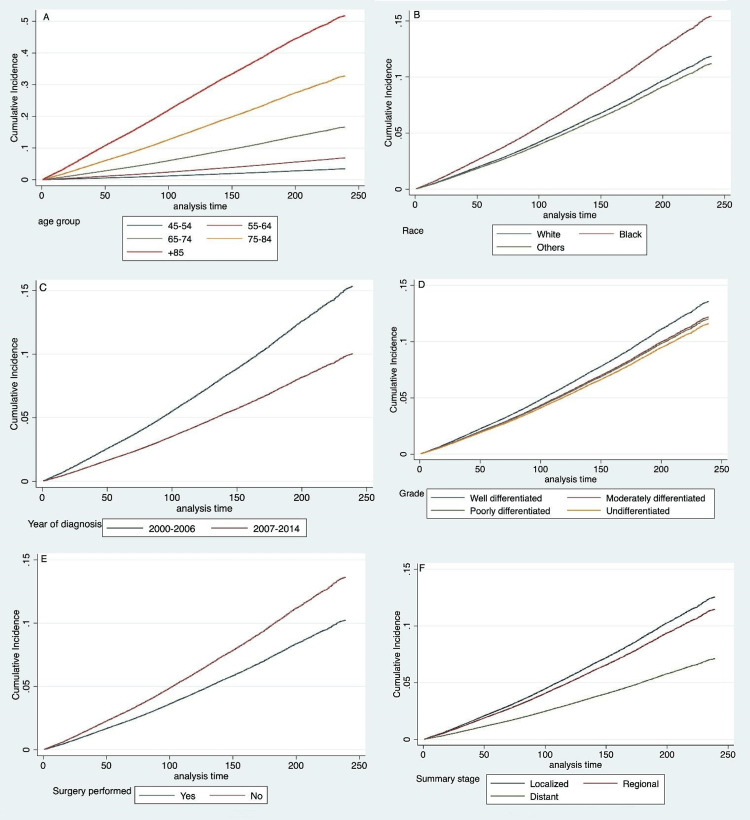
Cumulative incidence function curves for various factors of CVD mortality in prostate cancer patients: age group (A), race (B), year of diagnosis (C), grade (D), surgery performed (E), and summary stage (F) CVD: cardiovascular disease; NB: time in months

In our study, CVM risk was evaluated, and several indicators were found to be associated with a worse prognosis. The results revealed that among the five age groups, those 85 and older were the most susceptible to CVD death (SHR (95% CI): 20.9 (18.628-23.467)). In addition, it was observed that the black race positively predicted CVD death (SHR (95% CI): 1.3 (1.264-1.398)). Patients who did not undergo surgery were also found to be at a greater risk of CVD death (SHR (95% CI): 1.35 (1.305-1.410)). On the other hand, the variables that were found to be associated with a lower risk of CVD events included being of Asian/Pacific Islander or American Indian/Native Alaskan ethnicity (SHR (95% CI): 0.94 (0.891-0.993)), being diagnosed between 2007 and 2014 (SHR (95% CI): 0.63 (0.613-0.655)), having an undifferentiated (anaplastic) histology (SHR (95% CI): 0.84 (0.62-1.15)), and having a distant stage of the disease (SHR (95% CI): 0.54 (0.50-0.60)). Figure 2 shows the cumulative incidence function (CIF) curves.

## Discussion

This population-based analysis demonstrates that non-cancer mortality, of which CVD is the primary cause, accounts for a significant proportion of deaths in male PC patients. However, this finding is consistent with prior population-based studies conducted on patients with PC [24,25]. In our study, CVD death even outpaced mortality from PC [26,27]. A possible explanation is that men with PC may be at an increased risk of developing CVD due to the adverse effects of several medical treatments, such as androgen deprivation therapy, which may lead to an increase in blood sugar and cholesterol levels [28]. Patients with PC may also be more likely to have other CVD risk factors, including obesity, hypertension, and diabetes [29,30].

The Fine-Gray competing risk model in our study revealed several risk factors that are associated with poor prognosis in the study sample, including a stepwise increase in age, black ethnicity, and patients without surgical intervention. Satariano et al., in their study, found that, with increasing age at diagnosis, there was a greater possibility of dying from an illness other than PC [31]. Furthermore, age is a well-established risk factor for CVD, and, unsurprisingly, an increase in age would be associated with an increased risk for CVD in PC patients [32]. Moreover, studies have shown that black males have a higher chance of mortality from CVD than whites [33-35], and hence, their risk of developing PC is greatly increased. Additionally, non-surgery individuals may have other health issues that increase the incidence of death from CVD. For example, patients with PC who also suffer from other chronic conditions, such as diabetes or hypertension, may be at higher risk of CVD.

On the other hand, patients diagnosed between 2007 and 2014 reported a decreased risk of experiencing CVD mortality. Advancements in diagnostic and treatment procedures have probably resulted in better overall management of PC, resulting in a lower incidence of CVD among people with the condition [36]. In our study, patients with undifferentiated or distant PC stage have a lower incidence and a higher survival rate in terms of CVM than those with well-differentiated or localized PC in terms of CVD. A previous study suggests that the patient's likelihood of dying from cancer increases proportionately with the advanced stage of the disease at the time of diagnosis [37]. Also, it has been shown that those patients suffering from localized cancer are more likely to die of causes other than cancer itself if they have a greater number of comorbid conditions [38]. Van Hemelrijck et al. and Ketchandji et al. evaluate the causes of death in patients with PC [27,39]. According to their findings, CVD and other cancers are the main causes of death in men with early-stage PC and low-to-moderate-grade tumors.

According to our study, male patients with PC have a significantly increased risk of CVM compared to the general population. Our study used SMRs to compare the observed number of deaths from CVD in the study population to the expected number of deaths in a general US population. The results suggest an increase in the risk of death from CVD starting at 45-54 years old and continuing to decrease with lower SMR values in the older age groups, which is consistent with previous studies [8,40,41]. In addition, SMRs for CVD were substantially higher in Asian/Pacific Islander and American Indian/Native Alaskan populations than in white or black populations. A prior study using data from the SEER database found that death from reasons other than PC, in which CVD was the commonest cause, was higher in other ethnic groups than in white or black patients, which is consistent with our findings [26]. This finding supports the need to improve cardiovascular treatment and prevention for these patients.

Our retrospective analysis has some limitations that should be reported. Data aspects may be limited since the SEER database only gathers data on demographic, diagnostic, and treatment information and excludes other vital patient data such as lifestyle factors, medical history, and comorbidities such as prior CVD, diabetes mellitus, and dyslipidemia. Additionally, there may be differences in the reporting standards and practices of participating registries, which may impact SEER's accuracy and completeness of data. However, the SEER database does not give a representative sample of the total US population and only includes data from a few selected geographic areas; therefore, some selection bias may be present.

These limitations are crucial and should be carefully considered when interpreting the study's implications for clinical practice. Despite these constraints, our findings emphasize the pressing need for healthcare professionals to adopt strict management and monitoring strategies for the cardiovascular health of patients with PC.

## Conclusions

This study suggests that patients diagnosed with PC may have a significantly increasing risk of dying from CVD compared to the general population. Several key risk factors may appear to contribute to the incidence of CVD, including advanced age, being of black ethnicity, and not receiving a surgical intervention.
